# Evaluation of the effects of the powder of *Capsicum frutescens* on glycemia in growing rabbits

**DOI:** 10.14202/vetworld.2016.281-286

**Published:** 2016-03-16

**Authors:** Tossou Jacques Dougnon, Messanvi Gbeassor

**Affiliations:** 1 Laboratory of Research in Applied Biology, Polytechnic School of Abomey-Calavi, University of Abomey-Calavi, 01 BP 2009 Abomey-Calavi, Benin; 2 Faculty of Sciences, University of Lomé (Togo), BP 1515, Lomé, Togo

**Keywords:** blood sugar, *Capsicum frutescens*, consumption index, rabbit

## Abstract

**Aim:**

The present study aims to evaluate zootechnic parameters and blood sugar in rabbits submitted to diets containing different levels of pepper (*Capsicum frutescens*).

**Materials and Methods:**

To this end, 30 rabbits weighing on average 1252±35 g at the beginning of the experiment were subjected to five rations with three repetitions for 56 days: The food R0 (or control) which is floury provender contains 0% of *C. frutescens*; R5, R10, R15, and R20 provender containing, respectively, 0.5%, 1%, 1.5%, and 2% of *C. frutescens* fruits’ powder. Rabbits consumed on average from 75.47 to 80.97 g dry matter.

**Results:**

Digestibility ranged from 52.39% to 61.01%. The average daily gain and feed consumption index were similar for all diets. Blood glucose was amended by the various servings is 0.98 g/L and 0.88 g/L, respectively, for doses.

**Conclusion:**

It appears from this study that rabbits consumed well diets containing *C. frutescens*. However, *C. frutescens*’ effect on the growth performances of rabbits is not noticeable. Furthers experiments will be useful to evaluate *C. frutescens*’ mechanism of action on blood sugar.

## Introduction

Food self-sufficiency is one of the key priorities of developing countries in general and in Benin in particular. In fact, meat consumption (13 kg/person/year) in Sub-Saharan countries is below the average recommended by FAO (21 kg/person/year) [1]. Despite this, in the mind of consumers, food should no longer only cover nutritional needs but must contribute to the maintenance of human health [2]. However, it is noticed that one of the main causes of human diseases is malnutrition. Some diseases such as diabetes are major threats [3,4]. In Benin, its prevalence is 10 per 10,000 with 1.6% of deaths and lethality of 12.5% [5]. In fact, diabetes comes from a metabolic disorder caused by the body’s inability to produce insulin, a hypoglycemic hormone secreted by the pancreatic β-cells of the islets of Langerhans [6]. Currently, diabetes therapy is based on the use of hypoglycemic agents (sulfonylureas, biguanides, and insulin), on lifestyle changes, exercise, and requires lifelong treatment [7]. But, in Benin, due to the lack of access to health facilities and customary beliefs, most of the patients used exclusively or partially to traditional medicine.

In rabbits, the real diabetes is a very rare condition and is barely described in the scientific literature, with the exception of experimentally induced diabetes. When it occurs during the first phase of the disease, rabbits are able to compensate the lack of insulin production by the pancreas. It seems indeed that insulin plays a less important role in rabbits and herbivores in general than in carnivores and men [8]. But it sit could be that diabetes acts on the evolution of rabbits without the knowledge of the producers. Existing types of therapy cannot be used by farmers because of their excessive cost. So, many plants possess hypoglycemic properties, which after ingestion can help rabbits to regulate his glycemia. *Capsicum frutescens* is a perennial growing to 1 m (3 feet 3 in) by 0.6 m (2 feet). The fruits contain 0.1-1.5% capsaicin. This substance stimulates the circulation and alters temperature regulation. The seed contains capsicidins. These are thought to have antibiotic properties. However, these plants are not exploited because their food values are not well known [9].

This study aims to evaluate the effects of the powdered fruit of *C. frutescens* on glycemia in growing rabbits. Before we choose our experience doses of *C*. *frutescens* (0.5%, 1%, and 1.5%), we have made another experience with three diets containing 2.5%, 3%, and 3.5% of *C*. *frutescens*. This experience lasted 30 days and was conducted on 20 rabbits per diet. At the end, we noticed that these doses of *C. frutescens* (2.5%, 3%, and 3.5%) in the diet cause behavior troubles on rabbit’s body. This justifies the reason why we choose these doses of *C. frutescens* (0.5%, 1%, 1.5%, and 2%) to conduct our experience, and also as an author said, *C. frutescens* increased serum insulin concentration in a high-fat diet-fed streptozotocin-induced Type 2 diabetes rats after 4 weeks treatment.

## Materials and Methods

### Ethical approval

We note that all animals used for this research are with permission of the Animal Ethics Committee of University of Abomey-Calavi.

30 weaned rabbits with an average weight of 1252±35 g were used for this study.

### Powder of *C. frutescens*

Fruits of *C. frutescens* were obtained on the local market and dried in an oven at 65°C for 48 h to evaporate water completely. They were then reduced to powder in a mill (brand: RetschGmb H 5657 HAAN) at the Faculty of Agricultural Science of the University of Abomey-Calavi. An oven was used to determine the dry matter (DM) of prepared foods.

### Experimental design and experimental diets

The experimental design was a full randomized block design with five treatments (R0, R5, R10, R15, and R20). Each lot of animals was randomly assigned to one of five diets. Foods used are: The food R0 (or control), which is floury provender contains 0% of *C. frutescens*; R5, R10, R15, and R20 are floury provender containing, respectively, 0.5%, 1%, 1.5%, and 2% of *C. frutescens* fruit powder. Each diet is supplemented by oil palm leaves *ad libitum* will. The composition of the provender used as control food is presented in Table-1. The data of this study suggest that 2% dietary *C. frutescens* are insulinotropic rather than hypoglycemic in the experimental methods [10].

**Table-1:** Control diet composition.

Raw materials	%
Maize	5.0
Oil cake of palm tree	29.0
Cotton oil cake	16.0
Soya bean oil cake	7.0
Corn (wheat)	20.5
Rice	15.0
Shell of oyster	2.5
Salt	0.5
Sawdust	4.0
Total	99.5
Chemical composition	
DM (%)	88.4
Digestible energy* (MJ/kg of DM)	10.94
Cellulose (% of DM)	19.8
Total nitrogenous matter (% of DM)	18.8

* The digestible energy of food was obtained from the sum of the energy provided by each raw material entering its composition. Source: [11]. DM=Dry matter

### Conduct of the fattening

The experiment lasted 56 days and was conducted on rabbits of average live weight 1252±35 g. 100-130 g DM of food has been distributed to each rabbit every day from the beginning to the end of the test. The water was distributed *ad libitum*. Animals were weighed at the start of the test and every 14 days. The quantities of food offered and refused were recorded every 14 days. Evaluation of growth performances focused on the following parameters.

### Average daily gain (ADG)

ADG was calculated per animal and per period of 14-day following formula:

ADG = (Wd_14_−Wdi)/14 with Wdi: Weight at day i, and Wd_14_: Weight after 14 days of feeding.

### Consumption index (CI)

The CI is the amount of food consumed by an animal in kilograms to win a kilogram of live weight. CI was calculated for periods of 14-day following the formula below:

CI = I_d14_/ADG_d14_

Where, I_d14_ is the amount of DM (g) consumed in 14 days and ADG_d14_ is the average daily weight gain (g) in 14 days of feeding.

### Digestibility study

The digestibility study was conducted at 42 days of the fattening stage on 15 rabbits with a mean weight of 1615.03 g and divided into five homogeneous lots. The experimental design is the randomized complete block design with five treatments (R0, R5, R10, R15, and R20) which are the five food rations and three repetitions representing rabbits. Animals were placed in individual digestibility cages of 0.202 m^2^ area and 0.32 m height. These individual digestibility cages have drinking trough, a manger, and an alluvial collection system. The amount of food served, rejected, and droppings were weighed daily per animal. These samples were dried in an oven at 65°C for 48 h to determine the DM. The experiment lasted 7 days and at the end, the animals were weighed. The quantities of food distributed daily to each rabbit during this period were 100 g DM of feed and 20-30 g DM oil palm leaves.

### Total DM of food consumed

The amount of DM intake (DMI) per animal was calculated using the formula:

DMI = DMS−DMR with DMI the quantity of food ingested (in g of DM), DMS the quantity of food served (in g of DM), and DMR the food portion refused (in g of DM).

### DM digested food

Knowing the amount of food ingested we can determine the amount of digested food.

DMD = DMI−D with DMD the quantity of food digested (in g of DM), DMI the quantity of food ingested (in g of DM), and D the quantity of droppings (in g of DM).

The coefficient of apparent digestibility (CAD) in total DM was determined as follows:

CAD = 100 × DMD/DMI with DMD the quantity of food digested (in g of DM) and DMI the quantity of food ingested (in g of DM).

### Evaluation of glycemia in rabbits

Blood samples were collected in dry tubes from the auricular vein from 30 rabbits at the end of the experiment (after 56 days of feeding). Animals were subjected to 12 h of fasting before sampling. Samples were taken in the morning early at 7 am 30 min using a gauge needle Venoject. Samples were centrifuged for 5 min at 3200 tour/min, to obtain serum. Glucose rate was determined by spectrophotometry, respectively, with BIOLABO and ELI-tech kits.

### Statistics analysis

The software Statistix 8.0 served for statistical analysis. An analysis of variance (ANOVA) with two factors in the procedure of generalized linear models was used to examine the effects of different diets (R0, R5, R10, R15, and R20), measuring periods (n=4: 14; 28; 42; 56 days) and their interactions on dietary intake, ADG, and feed CI. Another one factor ANOVA was used to examine the effects of food intake on the biochemical parameter. The values presented were expressed by assigned average standard error of the mean. In the case of significant difference, Tukey HSD test was used to separate homogeneous groups at a significance level of 5%.

## Results

### Apparent digestibility and production droppings

Dietary intakes of DM ranged from 88.43 to 100.97 g/day (Table-2). DM droppings daily production in rabbits is similar for all diets and ranged from 38.08 to 46.22 g/day. DM digestibility ranged from 52.39% to 61.01% and was similar for the different diets.

**Table-2:** Change in digestibility and production droppings in rabbits.

Diets	Parameters		

	Ingested (g DM/day)	Droppings (g DM/day)	CAD (% DM)
R0	97.00±6.12	46.22±13.22	55.36±6.84
R5	88.43±6.12	41.60±13.22	52.39±6.84
R10	93.26±6.12	38.08±13.22	60.11±6.84
R15	100.97±6.12	42.26±13.22	58.48±6.84
R20	99.86±6.12	38.91±13.22	61.01±6.84
p	0.12	0.44	0.11

p=Probability of significance only, DM=Dry matter, CAD=Coefficient of apparent digestibility

### Food consumption and weight gain

Daily consumption of food by diet is presented in Table-3. The total consumption of food in MS during the test ranged from 75.47 to 80.97 g in rabbits. Body weights of the rabbits at the beginning of the trial was similar (p>0.05), and no significant differences were noted on the sharp end weight. The vivid average weight gain and the average CI are similar for different rations.

**Table-3:** Average consumption and CI.

Parameters	R0	R5	R10	R15	R20	p
Average consumption (g/MS)	76.22±7.91^a^	75.73±7.91^a^	75.47±7.91^a^	80.97±7.91^a^	77.66±7.91^a^	0.1789
Initial body weight (g)	1276.3±78.39^a^	1224.3±78.39^a^	1231.3±78.39^a^	1265.0±78.39^a^	1261.5±78.39^a^	0.954
Final body weight (g)	1952.7±113.59^a^	1897.3±113.59^a^	1903.2±113.59^a^	2078.0±113.59^a^	1976.0±113.59^a^	0.519
Daily weight gain (g)	12.07±26.89^a^	12.01±26.89^a^	11.99±26.89^a^	14.607±26.89^a^	12.75±26.89^a^	0.3243
CI	6.64±87.45^a^	9.92±87.45^a^	6.83±87.45^a^	5.95±87.45^a^	6.58±87.45^a^	0.5680

CI=Consumption index, the average in the same line followed by the same letter are not different significantly

**Figure-1:**
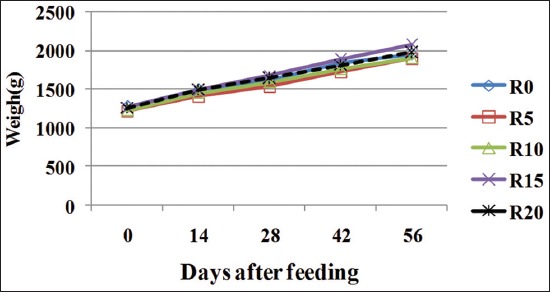
weigh evolution on rabbit

Figure-1 shows the weight change of the animals during the experimental period. It showed that at the beginning of the weights of the test batches are the same (p>0.05). Rabbits showed a linear increase regardless of the diet. At the end of the test, the body weights of the animals ranged from 1897 to 2078 g. No significant difference was observed between the final weights in rabbits receiving different rations of *C. frutescens* (p>0.05). Food CI ranged from 5.95±1 to 9.92±1 and was similar for the different food rations (Table-3).

### Evolution of feed intake, ADG, and feed efficiency

The evolution of food intake and weight gain of rabbits was presented in Table-4. Food intake, body weight, weight gain, and feed efficiency recorded in rabbits fed with rations with or without *C. frutescens* increased significantly from one period to another during the test (p<0.05). For the same period of feeding, dietary intakes of feed were similar. In the same period, the ADG and CI were similar. The interaction diets period was not significant for the following settings (p>0.05).

**Table-4:** Evolution of the food intake and the CI.

Parameters	Periods	Diets					Statistical variation source		
	
		R0	R5	R10	R15	R20	Diet	Period	Diet×period
Feed intake (g DM)									
	P14	79.06±3.13^aA^	81.166±4.44^aA^	80.93±2.07^aA^	80.76±8.20^abA^	78.94±4.81^abA^			
	P28	71.60±3.13^cA^	71.82±4.44^bA^	74.20±2.07^bcA^	77.68±8.20^cA^	76.94±4.81^bA^	0.178	0.000	0.842
	P42	78.40±3.13^abA^	81.64±4.44^abA^	79.01±2.07^abA^	87.03±8.20^aA^	81.93±4.81^aA^			
	P56	75.79±3.13^bA^	68.29±4.44^cA^	67.72±2.07^cA^	78.41±8.20^bA^	72.84±4.81^cA^			
ADG (g)									
	P14	15.38±1.56^aA^	13.61±3.28^aA^	16.07±2.35^aA^	16.07±3.20^bA^	16.44±3.10^aA^			
	P28	9.19±1.56^cA^	8.97±3.28^cA^	9.833±2.35^bcA^	11.94±3.20^cA^	11.02±3.10^cA^	0.324	0.000	0.9864
	P42	13.00±1.56^abA^	13.28±3.28^abA^	12.11±2.35^bA^	16.47±3.20^aA^	12.01±3.10^bA^			
	P56	10.73±1.56^bA^	12.19±3.28^bA^	9.94±2.35^cA^	13.58±3.20^bcA^	11.56±3.10^bcA^			
CI									
	P14	5.26±1.05^aA^	6.11±11.10^aA^	5.14±1.67^aA^	5.43±1.49^aA^	4.97±1.46^aA^			
	P28	8.12±1.05^aA^	21.79±11.10^aA^	7.95±1.67^aA^	7.10±1.49^aA^	7.82±1.46^aA^	0.568	0.1265	0.6614
	P42	6.07±1.05^aA^	6.15±11.10^aA^	6.58±1.67^aA^	5.39±1.49^aA^	6.86±1.46^aA^			
	P56	7.11±1.05^aA^	5.61±11.10^aA^	7.64±1.67^aA^	5.8±1.49^aA^	6.66±1.46^aA^			

The values followed by letter A on the same line are not significantly different, those followed by the different letters (a, b, c) on the same column are significantly different. DM=Dry matter, ADG=Average daily gain, CI=Consumption index

### Biochemical parameters

Assessing glycemia in rabbits is shown in Table-5. Blood glucose levels were significantly reduced for diets containing *C. frutescens*. It is lower in rabbits subjected to ration R15 and R20 but higher in rabbits subjected to R0 diet (p<0.05).

**Table-5:** Glycemia in rabbits.

Diets	Glycemia (g/L)
R0	1.15±0.02^a^
R5	0.98±0.02^b^
R10	0.97±0.02^b^
R15	0.89±0.02^c^
R20	0.86±0.02^c^
p	0.0000

Values followed by the different letters (a, b, c) on the same column are significantly different at 5% threshold.

## Discussions

### Growth performances

Ingestion of provender is very low in rabbits. This low consumption could be due to the floury presentation of the food which favored the sorting of the different food particles by animals. The results of Kpodékon *et al*. [11] showed that granulated feed provides significantly better performance in rabbits than floury ones. Furthermore, the rabbit poorly supports inevitably dust in the flour because it negatively impacts the normal operation of the nasal ways [12]. Similarly, researches of Goby and Rochon [13] on the digestibility and the impact of food sorting showed that rabbits forsake wood chips although rich in fibers.

The CAD of the registered DM is similar for the different tested food rations. Digestibility (DM) registered with the different diets is low compared with that (49.8-70.5%) obtained by Aboh *et al*. [14] with a ration based on the flour of *Mucuna pruriens* var. *utilis’* seeds supplemented with forages in rabbits. However, digestibility of diets used in this study is superior to that obtained (52.3% to 57.07%) by Dougnon *et al*. [15] in rabbits fed with pellets containing *Moringa oleifera* leaves. The daily production of droppings is greater than that (8.1-39.1 g DM/day) reported by Aboh *et al*. [14]. As against, it is less than that obtained by Dougnon *et al*. [15] using *M. oleifera* leaves (44.05-53.12 g DM/day). This difference is related to food ingredients and the physiological state of the rabbits because rabbits used had an average live weight of 1615.03 g against 1056.7±125.3 g and 2138.33 g, respectively, for those used by Aboh *et al*. [14] and Dougnon *et al*. [15]. The rabbits had a growth rate of 9.17-16.57 g/day during the test which is below the 22 g/day obtained by Kpodékon *et al*. [11], who used the same food formula. This difference could also be related to the form of presentation of the food and the physiological condition of rabbits used. Furthermore, Ludy and Mattes [16] suggested that capsaicin, the main constituent of *C. frutescens* can contribute to weight loss. However, the difference in weight observed in this study could not be attributed to this substance as the witness lot and those containing *C. frutescens* have similar weight gains. Conventionally, feed conversion ratio increases with the age of the animal [11]. The average CI obtained in this test is similar for all diets. The feed gain ratio (5.2:1) obtained during the last 4 weeks of fattening by Kpodékon *et al*. [11] with the same floury food is below that recorded during the study. This increase is related not only to the physiological condition of the animals used but also the presentation of the food because according to Kpodékon *et al*. [11], pelleted feed is consumed; less wasted and has a better feed efficiency than the food floury.

### Effect of *C. frutescens* on reducing glycemia through biochemical parameters

The results obtained reveal that glycemia level decreases with the increase of incorporation rate of *C. frutescens*. Post-prandial glycemia of control rabbits is 1.16 g/L. This result follows those of others studies where rabbits normal glycemia level is around 1 g/L [6,17]. The analysis of the results shows that, compared to the control lot, the consumption of *C. frutescens* produced variable effects according to the amounts applied for other animals. Indeed, at a low dose (0.5% and 1%), *C. frutescens* has a hypoglycemic activity and reduce glycemia level with a value of 0.98 g/L or a reduction of 15.51%. Between 1.5% and 2%, *C. frutescens* accentuates the hypoglycemic effect by reducing glycemia to 0.88 g/L or a reduction of 24.13%. These results can be explained by the chemical composition of *C. frutescens*, particularly capsaicin (with a rate of 0.1-1.5% in the plant) whose doses increased in relation to the doses of *C. frutescens* and consequently reduce glycemia level in rabbits. This result is similar to those of Chaiyasit *et al*. [18] which obtained in humans a reducing of glycemia level with 5 g of capsaicin, active ingredient of *C. frutescens*. In a similar way, [19] using fresh *C. frutescens* fruits in canids, obtain a reduction of glucose level and an increase of the insulin, a hypoglycemic hormone, level.

## Conclusion

From the results of this study, it appears that diets containing *C. frutescens* fruits powder were palatable by rabbits. The powder of *C. frutescens* had no effect on weight gain. However, powder of *C. frutescens* has a hypoglycemic effect which is accentuated when incorporation rate increases in food rations in rabbits.

## Authors’ Contributions

The present study was a part of original research work by TJD. He conceptualized the aim of the study, designed, planned, and supervised the experiment. Collection of samples and execution of experimental study were done by him and MG. Analysis of data, interpretation of the results, and drafting of the manuscript were done by TJD. MG helped in analysis, draft and revision of the manuscript. All authors read and approved the manuscript.
